# Movement competency in rowing: the key to an effective stroke

**DOI:** 10.3389/fspor.2025.1601563

**Published:** 2025-06-19

**Authors:** Natalie Legge, Katie Slattery, Mark Watsford, Damien O’Meara, Frank Nugent

**Affiliations:** ^1^ School of Behavioural and Health Sciences, Australian Catholic University, Strathfield, NSW, Australia; ^2^ School of Sport, Exercise and Rehabilitation, Faculty of Health, University of Technology Sydney, Moore Park, NSW, Australia; ^3^ Human Performance Research Centre, University of Technology Sydney, Sydney, NSW, Australia; ^4^ Sport Science Department, NSW Institute of Sport, Sydney, NSW, Australia; ^5^ Department of Physical Education and Sport Sciences, Faculty of Education and Health Sciences, University of Limerick, Limerick, Ireland; ^6^ Sport and Human Performance Research Centre, University of Limerick, Limerick, Ireland

**Keywords:** rowing, movement competency, physical attributes, stability, mobility

## Abstract

Movement competency combines fundamental patterns and movement quality that enables the confident and competent execution of activities, sports and everyday tasks. This perspectives article addresses the lack of a clear definition and guidelines relating to the sport-specific movement competency required for safe and effective rowing, particularly in the context of enhancing performance. In our opinion, movement competency should be emphasised together with the physiological and biomechanical attributes of rowing performance. Based on the literature, we have proposed the following definition, ‘*sport-specific movement competency for rowers incorporates the physical attributes of mobility and stability through the shoulders, trunk, hips, knees and ankles along with the associated muscular strength and endurance’ to coordinate and execute a technically effective stroke’*. Our definition highlights that rowers need to coordinate different regions of the body through appropriate joint positioning and movement patterns to safely optimise force development capacity during the stroke cycle. Examples of the mobility and stability requirements during the four main stroke phases are provided. The concept of sport-specific movement competency for rowing could provide benefits for rowing participation, technical rowing efficiency, injury prevention and performance enhancement.

## 1 Introduction

Movement competency refers to the fundamental patterns underlying movement that facilitates the confident and competent execution of activities, games, sports and everyday tasks (1–3). Developing appropriate movement competency early in the sporting pathway is critical to ensure physical readiness for the demands of sport (3, 4). The movement competency specific to a sport relates to a person's physical capacity to execute technique, whereby technique is defined as a coordination pattern that provides a movement solution specific to a sport (5). Movement competency in sport has largely been examined in relation to sports injury (4, 6); relationship with sport specialisation in youth populations (7); and association with the demands of particular sports (8). Despite a growing body of work, the literature is limited on the possible role of movement competency in the context of enhancing sports performance.

Sport-specific movement competency and enhanced performance outcomes have been documented for some sports. For instance, netballers who improved physical performance measures such as balance, agility and peak power after a 6-week neuromuscular training intervention also improved their movement competency through the assessment of a netball-specific movement screening tool (9). In a sporting context, competency across a range of movements has been recommended for safe, effective and long-term athletic development of young athletes (8, 10). Recent systematic reviews evaluating fundamental movement skills and movement competency in relation to sporting success have highlighted the need for clearer definitions and methods to define and measure sport-specific movement competence (11–13). Guidelines focussed on sport-specific movement competency are needed to provide benchmarks that reflect the movements of a certain sport (i.e., stability, mobility, balance, coordination or muscular strength) considered important to enable correct technique and safely meet the demands of the sport.

Essential movement competencies in rowing such as greater hip flexion, anterior pelvic tilt, trunk muscle strength and endurance have been highlighted in relation to injury (14). However, an all-encompassing term with a clear definition has not been established to reflect these attributes. Establishing a clear understanding of the movement competency requirements for rowing is needed, including quantitative performance-related benchmarks and guidelines for movement competency assessment specific to rowing. This has the potential to improve performance, reduce injury and retain participation in the sport. This perspective paper proposes the concept of movement competency specific to rowing.

## 2 Movement competency for rowing performance

Rowing is a technical sport that involves coordinating movements of the whole body and applying those movements to generate force on the oars and footplate that propel the boat forward (15). A definition for movement competency in rowing was informed by seminal articles identifying attributes that describe movement competency in rowing (14, 16). Potential attributes were condensed into key themes, and necessary attributes were then used to develop a preliminary definition (17). The preliminary definition was then refined in consultation with experts in rowing (18) to ensure internal consistency, simplicity and effectiveness. Following this process, our definition proposes that, ’sport-specific movement competency for rowers incorporates the physical attributes of mobility and stability through the shoulders, trunk, hips, knees and ankles along with the associated muscular strength and endurance to coordinate and execute a technically effective stroke’ (14, 19, 20). Mobility is defined as the range of movement (ROM) around a joint in combination with the associated flexibility which refers to the length of a muscle (21). Stability is defined as the restriction of joint movement controlled by several static and dynamic structures and mechanisms including ligaments and joint capsules, proprioceptive positional sense and muscular strength (22). Specific attributes of mobility and stability are required during each of the four main phases of the rowing stroke as outlined in the following section.

## 3 Main phases of the rowing stroke

**Figure 1:**
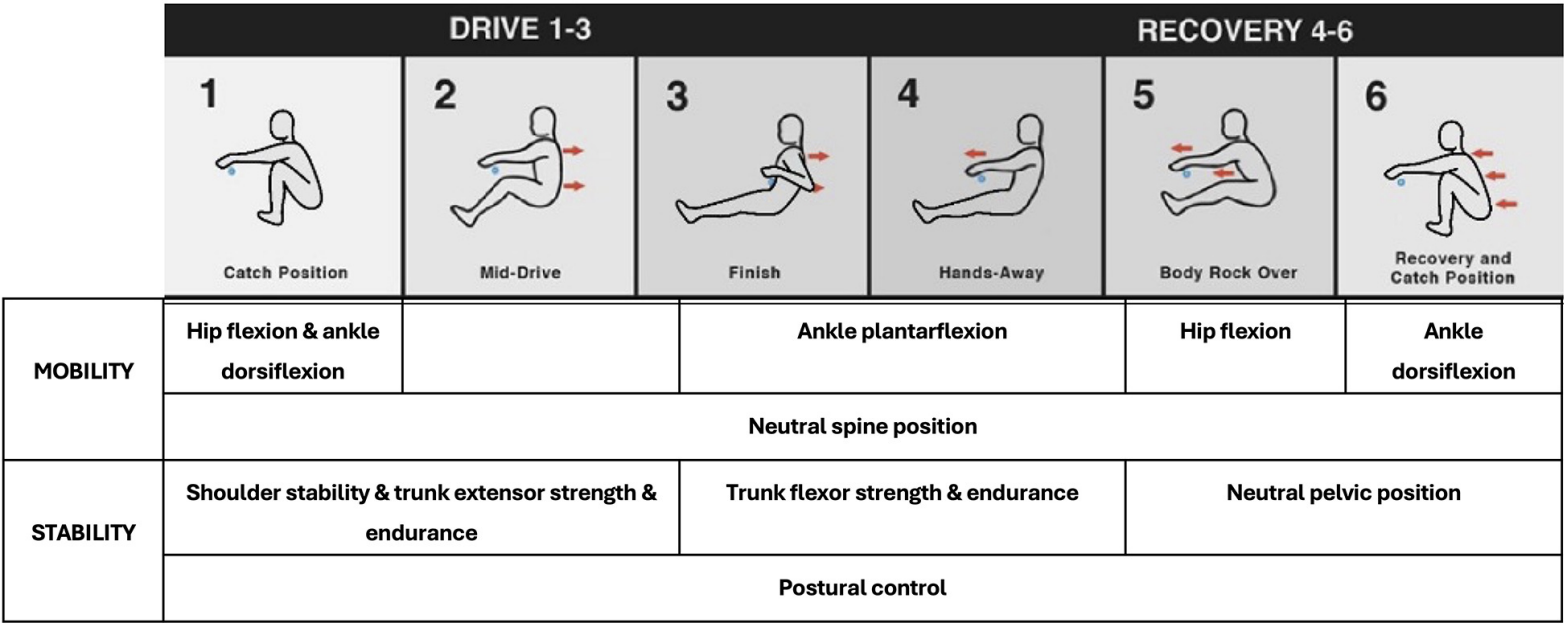
Proposed key mobility and stability attributes across the rowing stroke.

Rowing is a cyclical sport that involves the stroke being repeated over 200 times during a 2,000 m race. Given the high levels of repetition, movement competency in a cyclical sport like rowing may be of greater importance, in comparison to movement competency in field sports which have higher degrees of variability in movement such as jumping, catching, and tackling (23, 24). There are four main phases of the rowing stroke, the catch, drive, finish and recovery [Figure 1; (Legge et al., 2024)]. The “catch” position is the most unstable and technically challenging aspect of the stroke. It involves the blade being placed in the water and force rapidly developed to propel the boat forward (15, 25). This requires a position of full hip/knee flexion, and ankle dorsiflexion, while the spine remains neutral, and the upper limbs place the oar in the water with finesse to minimise disruption to boat momentum (26, 27). The “drive” phase involves knee, hip and trunk extension to transfer force from the foot-stretcher to the oar handle and blade in the water for forward propulsion. The “finish” completes the drive phase where the blade is extracted from the water in preparation for the “recovery” (27). The knees are fully extended at the finish position, the ankles are plantarflexed and the hips have finished extending; however, remain in a flexed position due to the upright seated posture (26, 28). The recovery is the non-propulsive phase of the stroke; however, it requires coordination and balance to mirror the sequence of body movements in the drive phase. The recovery is executed in the reverse order to the drive to initiate the optimal position for the subsequent catch. Rowing-specific movement competency requirements are specific to each of these four main phases.

### 3.1 Catch

The catch is a precise and challenging movement where the blade is placed in the water and force is rapidly developed to propel the boat forward (15, 25). To successfully execute this part of the stroke, an appropriate ROM to achieve the body position is required (28) alongside the associated force producing capabilities to maintain optimal posture for the development of boat propulsion (29, 30). With the legs and trunk producing 80% of rowing power (31), particular focus is required on the hips and trunk regions. Appropriate hip flexion has been reported in the range of 130° (28) and trunk stability required for rowing includes the muscular strength and endurance to maintain the required posture for the duration of a race (32, 33). Without these physical attributes, a rower may succumb to technical faults that are biomechanically inefficient and place undue repetitive loading through the lumbar spine/hips (14, 34).

Despite limited literature on the degree of ankle dorsiflexion required for an effective catch position and stretcher force application, an increase in passive ankle dorsiflexion range may enable a steeper foot-stretcher angle that can optimise propulsive force capabilities (35, 36). Conversely, if ankle dorsiflexion is limited, heel contact on the stretcher is reduced. To promote heel contact, the foot-stretcher angle and height may need to be adjusted, negatively impacting the ratio of horizontal to vertical stretcher forces and ultimately propulsive stretcher force (35, 37).

Shoulder stability is required at the catch as force is applied on the handle simultaneously with the foot-stretcher (Image 1, Figure 1). A stable shoulder girdle allows for more efficient transfer of force between the trunk and the oar handle (38). Chest wall injuries including rib stress injuries are common in rowing and although the aetiology is unclear (39, 40), excessive shoulder protraction can alter the balance with the shoulder retractors and lead to abnormal forces directed on the posterior aspect of the rib cage (41) and the serratus anterior and external abdominal oblique muscles may cause repetitive bending force to ribs (42). Addressing issues and establishing standards related to joint stability and muscle balance around the shoulder girdle and thoracic cage may positively impact both injury risk and performance. Accordingly, these should be considered an important aspect of movement competency for rowing.

### 3.2 Drive

The early to mid-drive phase is critical for a rapid rate of force development (43) and the lumbo-pelvic positioning should be relatively neutral with the primary movement generated through knee extension (38). The trunk acts as a lever throughout the drive phase and is the major link in the kinetic chain between the legs and arms (33). Trunk extensor muscle activity dominates up to 60% of the initial drive phase along with the hip extensors while trunk flexor activity is involved during the remaining 40%, contributing around the late drive and executing a braking action leading into the finish (8, 44). Strength training for rowing focuses on the drive phase given this is the propulsive phase of the stroke where peak force is achieved around the mid-drive (38, 45, 46). However, skillful rowers apply force earlier in the drive as well as maintain force for longer into the finish compared to less skilled rowers and this requires effective and coordinated movements from catch to finish each stroke (33, 45), highlighting the importance of movement competency.

### 3.3 Finish

The finish requires abdominal strength and endurance to maintain the trunk in a relatively neutral position and prevent posterior rotation of the pelvis which leads to excessive lumbar flexion (14, 47). At this stage of the stroke, the dominance of the trunk extensors and posterior chain muscles have transferred to the trunk flexors, acting as a brake to slow the trunk into the finish in preparation for the initiation of the recovery phase (33). Ankle plantarflexion around the finish has been suggested to increase stroke length and facilitates a smoother blade extraction from the water (36). However, passive ankle plantarflexion ROM is greater than that achieved during rowing therefore it is unlikely to be a limiting factor (36).

### 3.4 Recovery

The movement sequence from the finish to the recovery is typically described in coaching resources as commencing with the arms moving away from the body towards the stern of the boat, followed by a trunk rockover and lastly the hips and knees extending to move the seat forward towards the stern of the boat enabling the catch position (26, 27). Limited research has examined the recovery phase from a technical and physical perspective; however, coaches refer to attributes of coordination, balance and ‘boat feel’ when talking about effective recovery (48). Maximal velocity is achieved during the recovery phase, therefore there are two aims: to set up the body position for the next catch, and to minimise any disruption to the boat run during this process (27). Minimizing both intra-stroke and inter-stroke fluctuations in boat velocity has been associated with superior rowing performance and this stage of the stroke cycle is critical given there is no propulsive force application, and the body is moving against the direction of momentum (49).

The ability to ‘rockover’ through the hips is a key aspect of movement competency during the recovery phase (image 5, Figure 1). Therefore, hip mobility including hamstring flexibility is essential along with the trunk strength and endurance to maintain a neutral spine position. Excessive trunk flexion particularly in the lumbar spine may result as a compensatory movement due to lack of hip mobility (14, 50). When considering movement competency, this non-propulsive phase of the stroke cycle has the potential to provide gains in boat speed without greater physiological effort. Optimal body sequencing has been suggested by coaches as a key area for development in junior rowers to maximise boat run during this phase (48).

## 4 Movement competency screening in rowing

A key aspect of high levels of movement competency is the ability of the rower to coordinate different regions of the body through appropriate joint positioning and coordinated movement patterns to optimise force development capacity during the stroke cycle. Therefore, adopting a functional testing protocol specific to the movements comprising the rowing stroke as opposed to traditional athlete physical screening is needed (38). Physical screening is common in sport with traditional tests measuring isolated joint ROM, muscle strength and flexibility (51–53). However, more functional approaches evaluate an individual's physical attributes tailored to sport-specific requirements (20, 54). Rowing-specific limitations such as low hip flexion, ankle dorsiflexion or shoulder instability can increase risk of injury while also impacting performance. Accordingly, such attributes need to be assessed in an integrated manner reflective of the combined movement patterns of the rowing stroke (20, 38). Furthermore, pelvic and spinal mechanics can change during rowing with increased training durations and intensities. For example, it has been suggested that rowers without low back pain (LBP) display distinct kinematics to those that have experienced LBP (14). Therefore, it is important to consider an individual's movement competency when prescribing training and make adjustments based on known recommendations such as the maximal duration of ergometer prescriptions (14).

The functional movement screen (FMS^TM^) has been evaluated in relation to rowing injuries (55–57). Two studies examining seasonal data on collegiate rowers suggest the FMS^TM^ is not effective for injury prediction for rowing athletes (56, 57). For sport-specific movement competency, screening should reflect the movements, coordination and loading patterns of the sport. These studies reinforce the need for movement competency guidelines specific to rowing given the distinctive set of physical attributes specific to a rowing race, training demands, and the four main movement phases of the stroke cycle.

To maximise performance, minimise injury risk and to tolerate the demands of training and competition in rowing it is essential young athletes develop the necessary physical attributes (38, 48). This is where an awareness of movement competency can have an impact early in the sporting pathway. As an example, adequate muscular strength and endurance around the hip and trunk to enable maximal force transmission of the leg drive as well as sufficient mobility through the hips to achieve an optimal catch position are common attributes lacking in less skilled rowers and should be a key focus for addressing movement competency in development athletes (27, 48, 58). Moreover, trunk and scapular stability around the catch and finish positions are important physical attributes to optimise force development and decrease the likelihood of injury (59, 60). Common technical faults of a less skilled rower include incorrect sequencing of the body movements during the rowing stroke (48). This relates to movement competency when a lack of mobility and trunk strength and endurance are preventing the athletes from achieving the required positions to optimise their force development capacity (14, 33). Further recommendations for a rowing-specific movement competency screen should be developed and promoted within the rowing community.

## 5 Discussion

### 5.1 Practical applications & future perspectives

Strength and conditioning (S&C) programs such as those presented by Young et al. (38), Nugent et al. (26) and Rawlley-Singh (46) provide useful insights into training the movement competency and strength requirements for rowing. Further research that quantifies movement competency for rowing can support such programs and the development of evidence-based movement competency assessment tools will potentially have a greater impact and influence on training practices at all levels of the rowing community. Practical applications should involve implementing resources into rowing organisations and governing sporting bodies, particularly at school-age levels, where young rowers are prone to over-training, overuse injury and early dropout (58, 61). Incorporating movement competency requirements such as minimal benchmark standards for key movements, joint positions and associated screening tools for safe and effective rowing can provide positive outcomes that will safely improve rowing performance.

Assessment and management of an athlete's sport-specific movement competence requires multidisciplinary consideration, communication, and input (30). The physical therapist and S&C coach alongside the head coach can deliver an integrated approach to address each individual's movement competence and technical efficiency and incorporate these aspects into the on-land and on-water training program. We propose that establishing clear guidelines on movement competency for rowing can be beneficial for rowing participation, technical rowing efficiency, injury reduction and performance enhancement (26). More quantitative research is required to establish such guidelines in collaboration with some of the leading experts in rowing including coaches, S&C coaches, physical therapists, rowing biomechanists and applied researchers.

## 6 Conclusion

The purpose of this perspective paper was to present and describe the concept of movement competency specific to rowing. In our opinion, movement competency in rowing incorporates the physical attributes required to be able to execute a technically effective stroke through appropriate stability and mobility specific to rowing. It is clear that mobility and stability are required to achieve effective and coordinated positions throughout the rowing stroke cycle including the catch, drive, finish, and recovery to optimise performance and minimise injury.

## Data Availability

The original contributions presented in the study are included in the article/Supplementary Material, further inquiries can be directed to the corresponding author.
